# Seasonal variation of bacterial endophytes in urban trees

**DOI:** 10.3389/fmicb.2015.00427

**Published:** 2015-05-19

**Authors:** Shu Yi Shen, Roberta Fulthorpe

**Affiliations:** Department of Physical and Environmental Sciences, University of Toronto ScarboroughToronto, ON, Canada

**Keywords:** bacterial endophytes, endophytes, seasonal variation, species variation, trees

## Abstract

Bacterial endophytes, non-pathogenic bacteria residing within plants, contribute to the growth and development of plants and their ability to adapt to adverse conditions. In order to fully exploit the capabilities of these bacteria, it is necessary to understand the extent to which endophytic communities vary between species and over time. The endophytes of *Acer negundo, Ulmus pumila*, and *Ulmus parvifolia* were sampled over three seasons and analyzed using culture dependent and independent methods (culture on two media, terminal restriction fragment length polymorphism, and tagged pyrosequencing of 16S ribosomal amplicons). The majority of culturable endophytes isolated were Actinobacteria, and all the samples harbored *Bacillus, Curtobacterium, Frigoribacterium, Methylobacterium, Paenibacilllus*, and *Sphingomonas* species. Regardless of culture medium used, only the culturable communities obtained in the winter for *A. negundo* could be distinguished from those of *Ulmus* spp. In contrast, the nonculturable communities were dominated by Proteobacteria and Actinobacteria, particularly *Erwinia, Ralstonia*, and *Sanguibacter* spp. The presence and abundance of various bacterial classes and phyla changed with the changing seasons. Multivariate analysis on the culture independent data revealed significant community differences between the endophytic communities of *A. negundo* and *Ulmus* spp., but overall season was the main determinant of endophytic community structure. This study suggests studies on endophytic populations of urban trees should expect to find significant seasonal and species-specific community differences and sampling should proceed accordingly.

## Introduction

Bacterial endophytes are bacteria extracted from healthy looking, surface-sterilized plants (Hallmann et al., 1997). These bacteria can offer advantages to their hosts such as increasing nutrient acquisition including non-nodular nitrogen fixation, growth and development promotion through mechanisms such as production of growth factors, stress tolerance, pathogen and disease resistance, and contaminant degradation (Cook et al., 1995; Adhikari et al., 2001; Strobel et al., 2004; Moore et al., 2006; Ryan et al., 2008; Doty et al., 2009). The early literature on bacterial endophytes was dominated by studies on plants of agricultural importance such as rice (Sun et al., 2008), wheat (Conn and Franco, 2004), soybeans (Okubo et al., 2009), corn (Figueiredo et al., 2009), and potatoes (Garbeva et al., 2001). While agricultural species are still the main focus, a great deal of work on the endophytes of trees has been carried out, primarily because of the importance of trees for the production of biomass for biofuels and to phytoremediation efforts.

Because of their importance to both enterprises*, Populus* species have been heavily researched at the “microbiome” level (Hacquard and Schadt, 2015). Draft genomes have been prepared for several *P. deltoides* endophytes (Brown et al., 2012). In this group genetic markers indicative of plant senstive quorum sensing have been found (Schaefer et al., 2013). Taghavi et al. (2009) isolated 78 strains from poplar and willow trees, and sequenced four for genome characterization and gfp labeling—finding an *Enterobacter* sp st 638 that enhanced the growth of hybrid poplar cuttings. The fixation of nitrogen appears to be a common trait in endophytes. Doty et al. (2009) found numerous diazotrophic endophytes in *Populus trichocarpa* and *Salix sitchensis*. A diazotrophic and IAA producing *Burkholderia vietnamiensis* strain was isolated in *P. trichocarpa* (cottonwood), and its inoculation into Kentucky Bluegrass enhanced the grasses' nitrogen content and growth (Xin et al., 2009). More recently inocula derived from nutrient stressed poplar were shown to promote growth through N fixation (Knoth et al., 2014). Lodgepole pine seedlings (*Pinus contortia*) led to the isolation of *Paenibacillus polymyxa P32b-2R* that can fix nitrogen in pine and also western red cedar (Anand and Chanway, 2013; Anand et al., 2013). Other plant growth enhancing traits have been documented in poplar and other trees. Endophyte community structure seems to affect the ease of cultivation of *Prunus avium* (sweet cherry) cultivars (Quambusch et al., 2014). Mediterranean pines were found to harbor obligatory *Enterobacter cloacae*- some strains of which could produce indole-3-acetic acid (IAA) in lab culture (Madmony et al., 2005). Large portions of representative strains isolated from *P. euphratica* could enhance wheat germination under salt stress (Ju et al., 2014).

Endophytes that can assist plant growth on contaminated sites and that can contribute to the degradation of specific pollutants have been isolated from trees. The endosphere of *Acer pseudoplatanus* growing at a TNT contaminated site has yielded a consortium of strains (*Pseudomonas, Stenotrophomonas chelatiphaga*, and *Variovorax ginsengisol*a) that can detoxify TNT and promote growth of bent grass (*Agrostis capillaris*) (Thijs et al., 2014). *Enterobacter* sp st. PDN3 was isolated from hybrid poplar and shown to reduce TCE in the lab without any inducing substrate (Kang et al., 2012). van Aken et al. (2004) found a methane degrading methylobacterium (*Methylobacterum populi* sp. Strain BJ00) in hybrid poplar. Studies have shown that trees can act as hosts of degradative endophytes and/or degradative genetic material derived from elsewhere. The endophyte *Pseudomonas* sp. strain PD1 reduces phenanthrene toxicity via its degradation when inoculated into willows and grasses (Khan et al., 2014). Inoculation of poplar with *B. fungorum* DBT1 enhanced tolerance of the trees to PAH's (Andreolli et al., 2013). TCE remediation was enhanced in poplar by inoculation with *P. putida* W619-TCE, carrying a transferable plasmid that could move TCE metabolic activity to the endogenous endophytic population (Weyens et al., 2009). Taghavi et al. (2005) described the horizontal gene transfer of an introduced toluene degradation plasmid (pTOM-Bu61) amongst endophytic bacteria in a poplar strain, in both the presence and absence of toluene.

We were interested in looking for endophytic contributions to hydrocarbon degradation at a contaminated site in Toronto. Early reconnaissance of the site revealed the presence of numerous tree species and so we were first faced with practical questions such as how many tree species to investigate, how many individuals, and with what level of replication, and how often? But knowledge gaps exist in our understanding of the degree of host specificity found in endophytes—do plant species harbor specific endophytes, or can they act as hosts to any endophytic bacteria? How much do endophytic communities change over time? Studies on the determinants of endophyte community structure have focused typically on non-woody, agricultural plants and most of these involved the use of culture dependent methods (Adams and Kloepper, 2002; Kuklinsky-Sobral et al., 2004). Significant differences have been found in the endophytic community of different crop cultivars (van Overbeek and van Elsas, 2008; Manter et al., 2010). The diversity of bacteria in the grape endosphere has been shown to be highly dependent on season (Baldan et al., 2014; Bulgari et al., 2014), as have those of elm (Mocali et al., 2003). Contradicting results have been found by different authors, but these results were dependent on the methods used to analyze the communities. Izumi et al. (2008) found no difference in the endophytic communities of pine, birch and rowan trees in a European forest, although DGGE analysis was used that may have included organelle contamination (Izumi et al., 2008). By contrast, Ulrich et al. (2008) detected differences between the endophytic communities of poplar clones. Carrell and Frank (2014) found that pine and spruce species in the same nutrient limited environment had species specific endophytes communities, but nonetheless shared a dominant diazotrophic *Gluconacetobacter* species.

We undertook a characterization of the sources of variation in the endophytic communities of three tree species (*Acer negundo, Ulmus parvifolia*, and *Ulmus pumila*) growing at a hydrocarbon contaminated site. We do not report on the degradative or other functional traits of the endophytes here, but rather indicate the relative importance of species and season to endophyte community structure. This knowledge is key to (1) the design of good sampling strategies, (2) the prediction of the fate of introduced endophytes to different species, and (3) assess the fate of genetic material that may be expected to transfer into a dynamic community structure. To this end we used both culture dependent and independent methods.

## Materials and methods

### Sample collection and surface sterilization

Three branches from three individuals of each tree species—*A. negundo* (Manitoba Maple), *U. parvifolia* (Chinese Elm), and *U. pumila* (Siberian Elm) were collected in three seasons from an abandoned hydrocarbon contaminated site in Toronto, Ontario, Canada (43° 22′ 38″ N, 79° 18′ 34″ N). Samples were collected in February, July and October 2012, representing the seasons Winter, Summer and Fall, respectively. At the time of sampling, the outside temperature was as follows: Winter: −2°C, Summer: 22°C, and Fall: 11°C. Branches of roughly 1.5–2.0 cm in diameter were collected and the leaves and smaller branches were removed. The branches were subjected to a detergent wash, rinsed in tap and distilled water before being cut into sections ~ 9 cm in length. They were surface sterilized through successive washes in 70% ethanol, 0.1% Tween 20, and 1.5% bleach solutions. The sections were rinsed three times with sterile distilled water to remove any residual bleach. The effectiveness of the surface sterilization was assessed by spread plating the final water wash and imprinting the washed samples onto agar plates.

### Culturable endophyte extraction, isolation, and identification

#### Isolation of endophytes

The periderm of the sterilized branches was removed before the tissue was preweighed in a sterile tube, then homogenized in a sterilized Waring Blender jar at 20,000 rpm with 60 mL of 50 mM Tris-HCl solution for 1 min. The homogenized plant tissue was filtered through 8 layers of sterile cheesecloth and the liquid macerate was collected. The macerate was centrifuged at 600 g for 5 min to pellet plant tissue and the supernatant was centrifuged at 10,000 g for 10 mins to pellet the bacterial cells. The bacterial cells were resuspended in 1 mL of 50 mM Tris-HCl solution, and 100 μL was spread plated onto replicate Reasoner's 2A (R2A, Sigma-Aldrich Canada Co.) and Tryptic Soy Agar (TSA) media plates. The remainder of the macerate, including plant tissue, was used for DNA extraction. The media plates were incubated at 28°C for a period of 7 days to 1 month. For each branch processed, colony types were categorized by color/colony morphologies—each type was counted and a representative was taken for purification and identification.

#### Identification of isolates

Lysates were created from colonies of isolates by boiling. Approximately 2 uL of fresh (1–2 day old) colony material was added to 100 μL of sterile distilled water and boiled for 5 min. For each isolate, one microliter of the boiled lysate was used as a template for amplification of ribosomal 16S gene fragments (16S rRNA) using primers 27F (5′- AGAGTTTGATYMTGGCTCAG -3′) and 1492R (5′-TACCTTGTTACGACTT-3′). The PCR amplifications were carried out in a PTC-200 thermal cycler (MJ Research Inc.) with the following conditions: initial denaturation at 95°C for 5 min followed by 35 cycles of: denaturation at 95°C for 1 min, annealing at 56°C for 1 min and extension at 72°C for 1 min; final extension at 72°C for 10 mins. The amplicons were PCR purified using the GenElute PCR cleanup kit (Sigma-Aldrich Canada Co.) and sent to The Centre for Applied Genomics (TCAG,Toronto, Canada) for Sanger sequencing. The obtained sequences were submitted to the RDP database and BLAST (NCBI) in order to determine the potential identity of the bacteria based on minimum 99% similarity to database 16S rRNA sequences.

### Culture independent endophyte community extraction and analysis

#### DNA extraction of endophytic community

Plant macerates were used to extract total tissue DNA using the FastDNA SPIN Kit (MP Biomedicals) following manufacturer's instructions with a couple of modifications. The modifications included the addition of 100 μL of protein precipitation solution (PPS) solution into the lysing tube and 2 additional SEW-SM washes.

#### Enzymatic digestion, DGGE, PCR amplification, and T-RFLP analysis

Direct amplification of plant DNA tissue using universal bacteria primers produced amplicons dominated by plant organelle sequences (plastids and mitochondria). The primers 799F (5′-AACMGGATTAGATACCCKG-3′: Chelius and Triplett, 2001) and 783R (Sakai et al., 2004–an equimolar mixture of 783R-a (5′-CTACCAGGGTATCTAATCCTG-3′), 783R-b 5′-CTACCGGGGTATCTAATCCCG-3′), and 783R-c (5′-CTACCCGGGTATCTAATCCGG-3′), have been offered as a means of excluding plastids and mitochondria. Through preliminary testing we have found the effectiveness of 799F primer is dependent on the genotype of the plant and resulted in very minimal amplification of the bacterial sequences in our plant samples. This is similar to what has been previously found by Rasche et al. (2006) in their study of sweet pepper shoots. For this reason, an enzymatic digestion protocol was developed to try to bypass the problem of plastid contamination. Based on the fact that chloroplast 16S rRNA sequences have a *Pvu*II restriction site downstream of the binding site of 27F primer and a *Msc*I restriction site upstream of the binding site of 1492R primer, predigesting genomic DNA with those restriction enzymes should inhibit the amplification of plastid products from 27F to 1492R, allowing only the amplification of bacterial 16S rRNA and its subsequent analysis by molecular methods.

This method was initially tested on a couple of samples using DGGE as a preliminary visualization tool to determine how effective the method was. Using the previously extracted DNA from *A. negundo, U. parvifolia*, and *U. pumila* from Summer 2012, DNA from each sample was digested with the *Pvu*II and *Msc*I (NEB Canada). The samples were digested for 3 h at 37°C, followed by incubation at 80°C for 20 min to inactivate the enzymes. One microliter of the digested products was used as template for PCR reactions using primers 27F and 1492R following the previously mentioned conditions. The PCR products were visualized in 1.5% agarose gels and bands of 1500 bp in size were excised and gel purified with QIAEX II Gel Extraction Kits (Qiagen, Canada).

The gel-purified products were used as templates for the following PCR reaction using primers341F-GC (5′- CGCCCGCCGCGCGCGGCGGGCGGGGCGGGGGCACGGGGGGCCTACGGGAGGCAGCAG-3′) and MOD783R (equimolar concentrations of primer 783RA and primer 783RC- a modification of primers by Sakai et al., 2004). For the amplification, 20 μL PCR reactions were carried out in a PTC-200 thermal cycler (MJ Research Inc.) with the following conditions: initial denaturing at 95°C for 5 min followed by 35 cycles of: denaturing at 95°C for 1 min, annealing at 56°C for 1 min and extension at 72°C for 1 min; final extension at 72°C for 10 mins. The PCR products were checked on 1% agarose gels before the remainder of the PCR products was run in a DGGE gel. The DGGE ge lwas gel was a 6% polyacrylamide gel consisting of a 40–70% denaturing solution gradient and it was run in a DGGE-2001 Tank (C.B.S. Scientific Co, Del Mar, California) with 0.5 X Tris-acetate-EDTA buffer, for 20 h at 70V and 58°C. The gel was stained in ethidium bromide for 30 min before it was visualized under UV light.

Once the enzymatic predigestion was tested, subsequently for each sample, up to 1 μg genomic DNA was digested using 1U each of restriction enzymes *Pvu*II and *Msc*I for 16 h at 37°C. The digested genomic DNA was used as template for the 16S rRNA PCR reaction using a forward primer labeled with 5′-fluorescein amidite dye (27F-FAM from LifeTechnologies, Canada) and a reverse primer labeled with 5′–hexachlorofluorescein dye (1492R-Hex from LifeTechnologies, Canada). The PCR reactions were carried out in 20 μL reaction volumes following the previously mentioned PCR conditions. For T-RFLP (terminal restriction fragment length polymorphism) analysis the amplicons were digested with 1 U *Msp*I restriction enzyme (Thermo Scientific) for 3 h at 37°C followed by inactivation through incubation at 80°C for 20 min. The digested PCR amplicons were sent to the Agriculture and Food Laboratory (AFL) at the University of Guelph for analysis. Only fragments between 60–1200 bp with fluorescence signals greater than 100 units were included in the output from AFL. The Microsoft Excel macro Treeflap (Rees et al., 2004) from http://urbanstreams.net/index.php/the-treeflap-macro/, was used to round the fragment sizes to the nearest base pair and align the fragments of the same size from different samples, generating a cohesive table of the different fragments sizes and their relative heights in each sample. Fragments in the dataset in the range of 335–338 and 400–403 bp were assumed to represent potential mitochondrial fragments and plastid fragments and omitted from the data. Fragments sizes that had less than 1% abundance or that appeared in less than three samples were removed to account for any background noise generated during the PCR reaction, sequencing artifacts or rare members of the communities.

#### Pyrosequencing and analysis

For the pyrosequencing analysis of the culture independent community, equimolar subsets of the digested genomic DNA from the same plants and seasons were pooled together to create 9 plant/season samples. These 9 pools were submitted for 454 pyrosequencing using a Roche 454 FLX titanium instrument at MR DNA (Molecular Research LP) using barcoded facility primers 27Fmod (5′- AGRGTTTGATCMTGGCTCAG-3′) and 530R (5′- CCGCNGCNGCTGGCAC-3′). The data were analyzed using programs in the QIIME pipeline (Caporaso et al., 2010). Sequences with high error content were removed with Denoiser (Reeder and Knight, 2010), placed into OTU's of 97% similarity using UCLUST (Edgar, 2010) while omitting any reads belonging to mitochondria and chloroplast from the analysis. Sequences were rarified to 2000 per sample and the taxonomy was assigned through the use of the Greengenes Database (DeSantis et al., 2006) files from May 2013. The analysis of this data revealed that although this method allowed for the detection of bacterial species found in the samples, plastid 16S rRNA fragments were still amplified in the sample. We decided to try the plastid and mitochondrial oligonucleotide blockers designed by Lundberg et al. to inhibit the amplification of these organelle sequences in the sample (Lundberg et al., 2013). The blockers were used following their specifications. In brief, the oligonucleotide blockers were added to the PCR reaction at a final concentration of 0.5 μM, with primers 27F and 1492R. The PCR was carried out with the previously mentioned conditions with the addition step of 78°C for 30 s prior to the primer annealing step and the reaction was carried out for 25 cycles. The PCR amplicons were gel size selected (for ~1500 bp) and purified with QIAEX II Gel Extraction Kits (Qiagen, Canada). The amplicons from the same plants and seasons were pooled together and submitted for 454 pyrosequencing using the same machine and primers previously mentioned. This data was analyzed using the same method previously mentioned with the sequences rarified to 10,000.

### Community and statistical analysis

All of the community and statistical analyses were performed using packages in R 2.15.2 (R Core Team, 2012). The culturable community consisted of the bacteria isolated from the plant macerate and their respective abundances relative to the total amount of bacteria isolated from the same sample. Two communities were generated for each sample as the plant macerate was cultured on R2A and TSA media. The nonculturable community assessed through the use of T-RFLP, relied on the phylotypes in the form of different terminal fragment (T-RFs) detected by the autosequencer and their respective abundance in the sample. The species richness and the species diversity, based on the Shannon index, were determined for each sample. The homoscedascity and the normality of each variable was checked through the use of Levene's test and Shapiro's test (lawstat package, Noguchi et al., 2009). If the data were determined to be homogeneous and normally distributed, ANOVA (analysis of variation) tests were conducted. In the cases where the data were not homogeneous and/or normally distributed after transformation, Kruskal-Wallis tests were conducted. The culturable and nonculturable endophytic community compositions were compared between the different tree species and seasons by using Bray-Curtis dissimilarity matrices, non-metric multidimensional scaling (NMDS, ecodist package, Goslee and Urban, 2007) analysis and permutation multivariate analysis of variance (ADONIS) using functions found in the vegan package (Oksanen et al., 2012). ADONIS analyses were run with 1000 permutations.

## Results

### Culturable isolates and community analysis

We used two different media to represent both low nutrient (R2A) and rich media (TSA) to allow for the detection of a wide variety of bacteria. Through the culturing of the plant macerates on the two media, the total amount of culturable bacteria obtained from each plant sample per season ranged from 10^3^ to 10^7^ colony-forming units (cfu) per gram of fresh tissue. There were significantly higher total counts in all species in the Fall compared to the other seasons (*p* < 0.05). However, neither total bacterial counts, species richness, nor species diversities (Shannon Index) were different between media types (Supplementary Figure 1).

**Figure 1:**
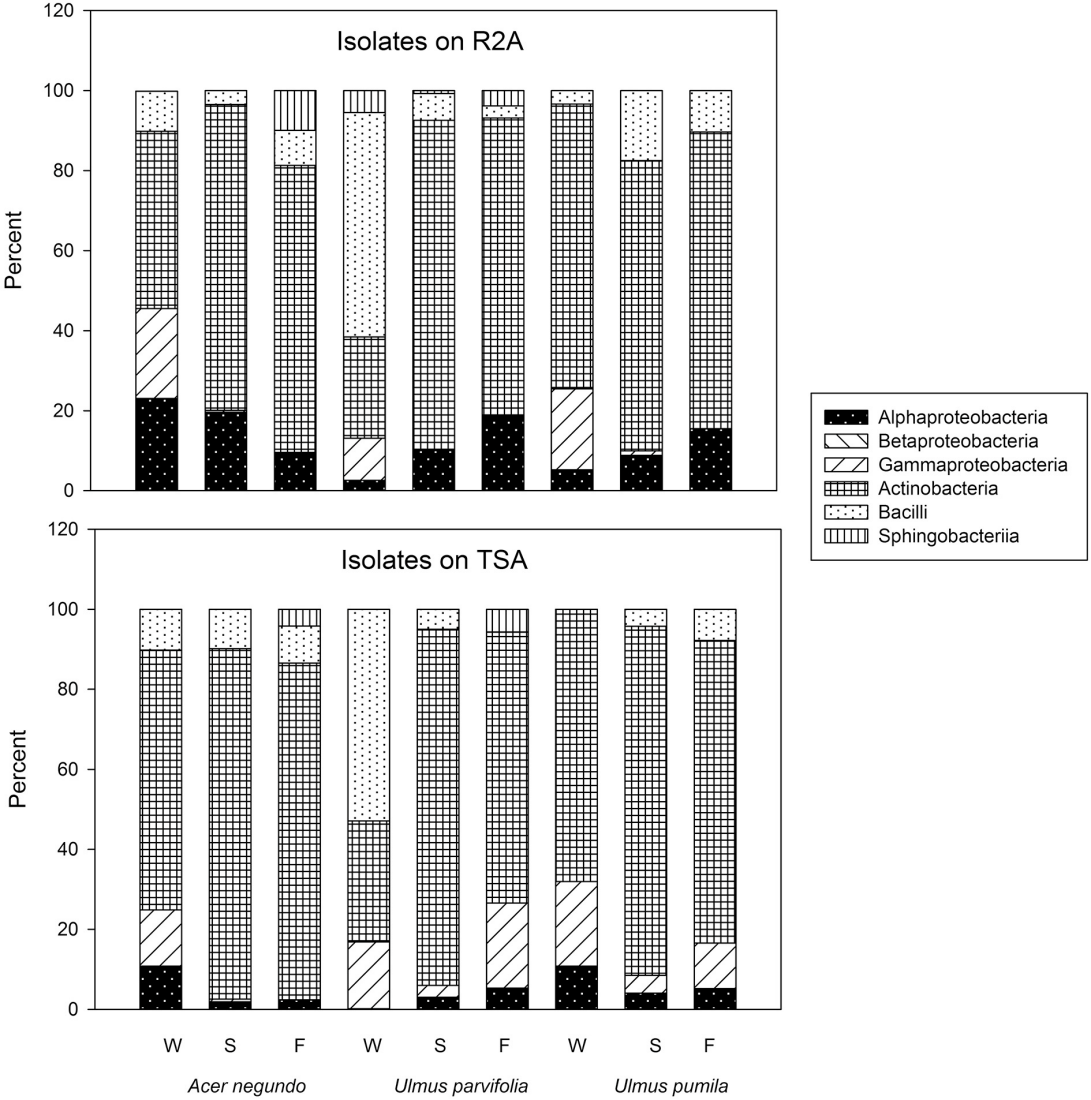
**Percent abundance of cultural bacteria in phyla/classes**. W, Winter; S, Summer; F, Fall.

A total of 31 different bacterial genera were cultured using the two media from all plant samples combined as shown in Table 1. *Variovorax* spp. and *Amnibacterium* spp. could only be isolated from *A. negundo* while *Rhizobium* spp. and *Rathayibacter* spp. were only isolated from *U. parvifolia*. Some bacterial genera were isolated from all the plant species, throughout all 3 seasons: *Bacillus* spp., *Curtobacterium* spp., *Frigoribacterium* spp. *Methylobacterium* spp., *Paenibacillus* spp., and *Sphingomonas* species. All of the genera identified belonged to one of the following phyla: Actinobacteria, Firmicutes, Bacteroidetes, Deinococcus-Thermus, and Proteobacteria. A breakdown of the relative abundances of the culturable endophytic isolates based on class per plant and season is shown in Figure 1. The majority of the isolates were from the phylum Actinobacteria, making up 62% and 63% of total bacterial isolated in R2A media and TSA media, respectively. Under the phylum Proteobacteria, bacteria specifically belonging to the class of Alphaproteobacteria, Betaproteobacteria and Gammaproteobacteria were found. Differences in the media used resulted in a higher percentage of Alphaproteobacteria isolated on R2A whereas a higher percentage of Gammaproteobacteria were isolated on TSA. It also made a difference in the actual isolates obtained as *Amnibacterium* spp., *Pseudoclavibacter* spp., and *Rhizobium* spp. were only cultured on TSA whereas *Deinococcus* spp., *Mesorhizobium* sp., and *Variovorax* spp. were only cultured on R2A. There were other noticeable changes in the cultured endophytes such as fewer Firmicutes and Gammaproteobacteria in the Summer and Fall relative to Winter samples, and an increase of Bacteroidetes bacteria cultured in the Fall. 16S sequence data for these isolates can be found in GenBank accessions KP889009-KP889059.

**Table 1:** **Cumulative list of identified bacterial endophytes and their corresponding bacterial class, isolated from *A. negundo, U. parvifolia*, and *U. pumila* branches from Winter, Summer, and Fall**.

**Bacterial Class**	**Bacterial Genera**	
Actinobacteria	*Agrococcus* sp. *Amnibacterium* sp.**^*T, A*^** *Arthrobacter* sp. *Brevibacterium* sp. *Curtobacterium* sp. *Friedmanniella* spp. *Frigoribacterium* spp. *Geodermatophilus* sp. *Kineococcus* sp.	*Kocuria* sp. *Microbacterium* spp. *Nocardioides* sp. *Patulibacter* sp.^*^ Plantibacter sp. *Pseudoclavibacter* spp.**^*T*^** *Rathayibacter* sp.**^*B*^** *Sanguibacter* spp.
Bacilli	*Bacillus* spp. *Paenibacillus* spp. *Staphylococcus* spp.	
Flavobacteriia	*Chryseobacterium* sp.	
Deinococci	*Deinococcus* sp**.^*R*^**	
Alphaproteobacteria	*Mesorhizobium* sp.^***R***^ *Methylobacterium* spp. *Paracoccus* sp. *Rhizobium* sp.^***T, B***^ *Sphingomonas* spp.	
Betaproteobacteria	*Variovorax* sp.**^*R, A*^**	
Gammaproteobacteria	*Pseudomonas* spp. *Stenotrophomonas* sp. *Xanthomonas* spp.	

* *Not previously mentioned in literature as endophyte. **T**, Isolated only from TSA media; **R**, Isolated only from R2A media; **A**, Isolated only from Acer negundo; **B**, Isolated only from Ulmus parvifolia*.

In addition to the bacteria that could be isolated and identified, there were a variety of bacteria that initially grew on the spread plates of the plant macerates but would not subsequently grow on subculture. These unidentified bacteria accounted for 6% and 13% of the total bacterial count isolated from R2A and TSA, respectively.

For both media, NMDS analysis resulted in visible clustering of *A. negundo* samples away from *U. parvifolia* and *U. pumila* samples in all seasons (Supplementary Figure 2). However ADONIS testing showed that only the Winter samples exhibited significant differences in the culturable community of *A. negundo* from *Ulmus* spp. (Table 2). The *U. parvifolia* community isolated in R2A media in each season was statistically different from the other seasons, whereas in TSA only the community in the Winter was statistically different from the other seasons. For *U. pumila* and *A. negundo* the Fall samples were found to be distinguishable from the other seasons.

**Table 2:** **Results of ADONIS test run on endophytic communities obtained from culture based methods (R2A and TSA) and culture independent method (T-RFLP)**.

	**Category**	**Subsequent testing**	***p*-value**	**Distinguishable component**
R2A	Season	*A. negundo*	0.001^*^	Fall
		*U. parvifolia*	0.001^*^	Winter, Summer Fall
		*U. pumila*	0.034^*^	Fall
	Plant	Winter	0.014^*^	*A. negundo*
		Summer	0.47	
		Fall	0.16	
TSA	Season	*A. negundo*	0.31	
		*U. parvifolia*	0.002^*^	Winter
		*U. pumila*	0.10	
	Plant	Winter	0.013^*^	*A. negundo*
		Summer	0.60	
		Fall	0.13	
TRFLP	Season	*A. negundo*	0.001^*^	Summer
		*U. parvifolia*	0.004^*^	Fall
		*U. pumila*	0.013^*^	Summer
	Plant	Winter	0.001^*^	*A. negundo*
		Summer	0.042^*^	*A. negundo*
		Fall	0.025^*^	*A. negundo*

Testing of significant difference determined through the use of pairwise comparisons and bonferroni correction.

* *Significant p-values*.

### Culture-independent community analysis

#### Use of predigested DNA samples for T-RFLP community analysis

**Figure 2:**
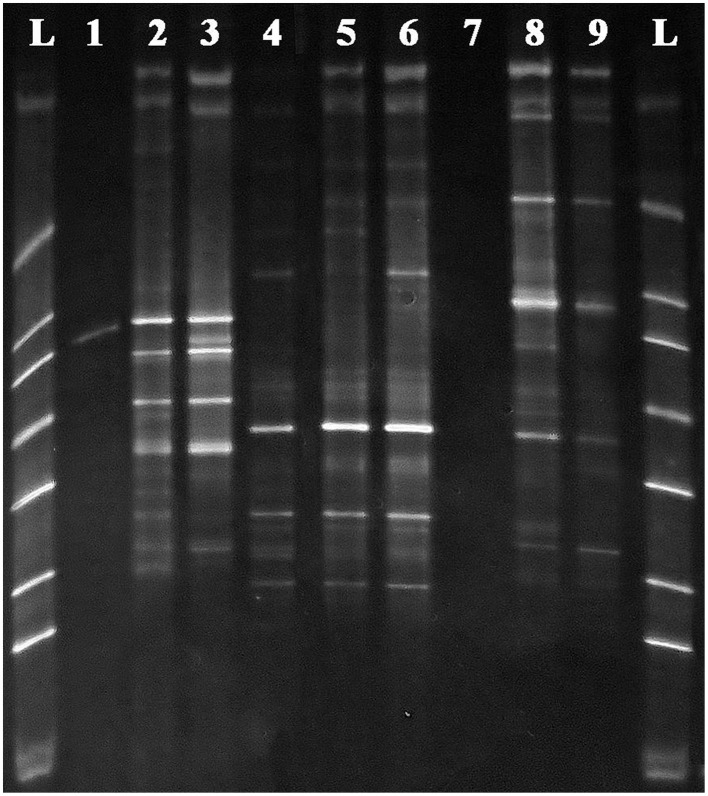
**DGGE gel of 341GC-MOD783R PCR amplifications on DNA samples from (1) original *Acer negundo* DNA, (2) *Msc*I digested *Acer negundo* DNA, (3) *Pvu*II digested *Acer negundo* DNA, (4) original *Ulmus parvifolia* DNA, (5) *Msc*I digested *Ulmus parvifolia* DNA, (6) *Pvu*II digested *Ulmus parvifolia* DNA, (7) original *Ulmus pumila* DNA, (8) *Msc*I digested *Ulmus pumila* DNA, (9) *Pvu*II digested *Ulmus pumila* genomic DNA and L–DGGE ladder**. Note products are all bacterial due to use of 783 primer, but are not well amplified from undigested targets.

Initial testing of the enzymatic predigestion of total DNA was originally conducted on extracted DNA from the three plant species examined in this study. The results of this testing are shown in Figure 2, which shows a DGGE analysis of the amplicons obtained from undigested original samples and those digested by either *Pvu*II or *Msc*I using the primers 341GC-MOD783R that do not target organelles. The figure demonstrates that bacterial fragment amplifications were poor prior to pre-digestion of the template, presumably because of organelle DNA dominance in the samples. The number of bacterial bands detected was either the same as the original undigested samples or in cases where there was minimal to no amplification, it improved the amplification of bacterial sequences present in the sample.

#### T-RFLP community analysis

The examination of the T-RFLP communities resulted in the detection of mean of 27 (+/- standard deviation of 14) phylotypes per sample. These phylotype numbers should be assumed to be underestimates or minimums, as the removal of small fragments and fragments with small peak heights might have deleted data on more than just artifacts. Data on phylotype richness and diversities are shown in Supplementary Figure 3. There were 5 phylotypes found to be common to all branches sampled. The sizes of the phylotypes were as follows: 61 bp, 119 bp, 128 bp, 488 bp, and 612 bp.

**Figure 3:**
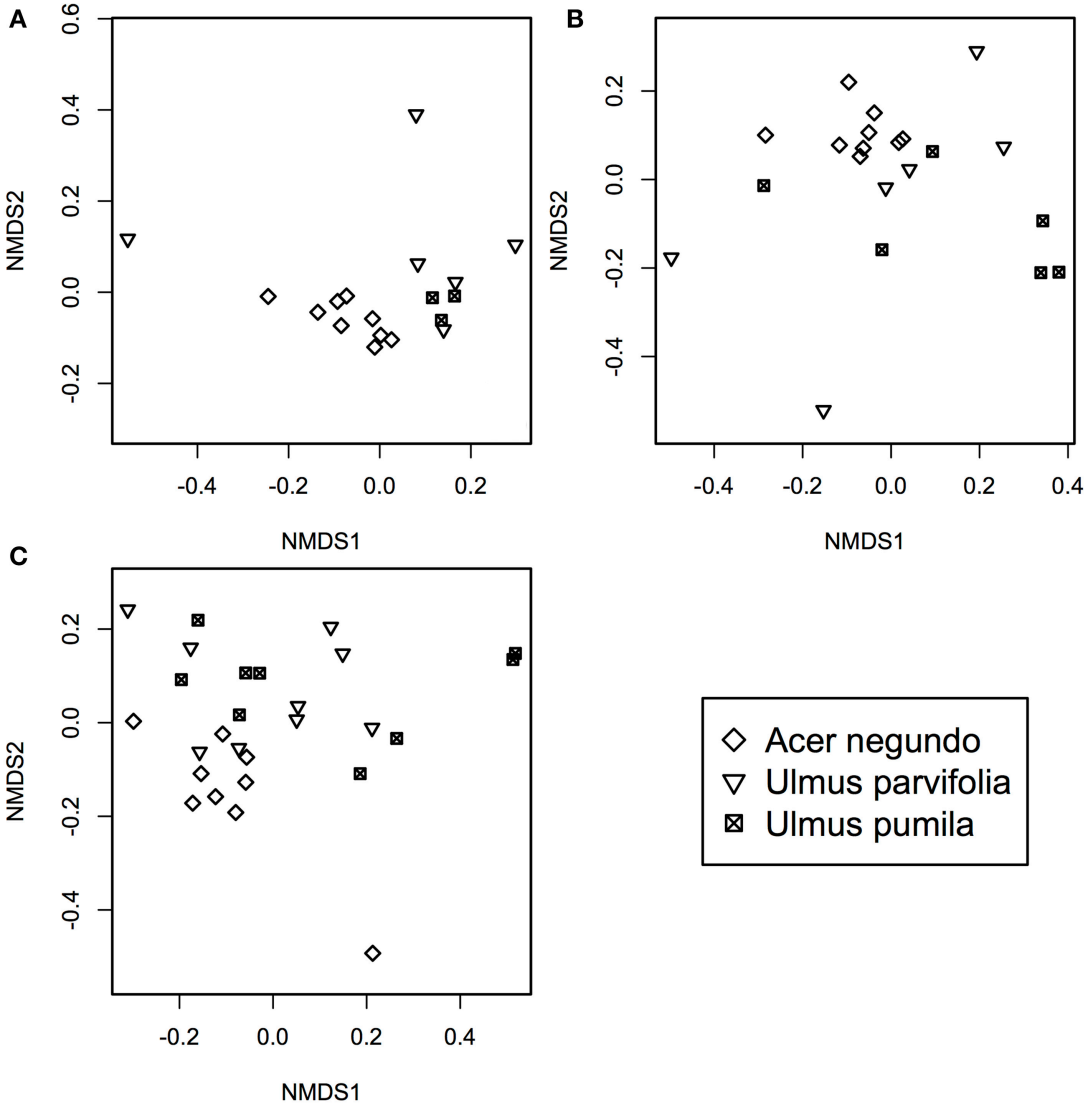
**NMDS plots of bacterial community profile of *A. negundo, U. parvifolia*, and *U. pumila* generated using T-RFLP community data converted to a Bray-Curtis dissimilarities matrices for (A) Winter 2012 (stress value = 0.05), (B) Summer 2012 (stress value = 0.04), and (C) Fall 2012 (stress value = 0.10)**.

*A*. *negundo* samples had higher phytotype richness than *Ulmus* samples (*p* < 0.05). NMDS and ADONIS analysis of T-RFLP based community compositions showed that regardless of the season the samples were from, *A*. *negundo* samples were always statistically distinct from those of *U*. *parvifolia* and *U*. *pumila* (Table 2, Figure 3). However no significant differences were found between the communities of *U*. *parvifolia* and *U*. *pumila* samples in any season.

When grouped according to their plant species, the NMDS analyses showed that Summer samples typically clustered apart from those from Winter and Fall (Supplementary Figure 4). The ADONIS test showed that there was a significant difference between the community profiles of the samples collected in the Summer compared to those collected in Winter and Fall for the *A*. *negundo* and the *U*. *pumila* samples (*p* < 0.05). For *U*. *parvifolia*, it was found that the community profiles of the samples collected in the Fall were significantly different from those collected in Winter and Summer.

#### Pyrosequencing data

**Figure 4:**
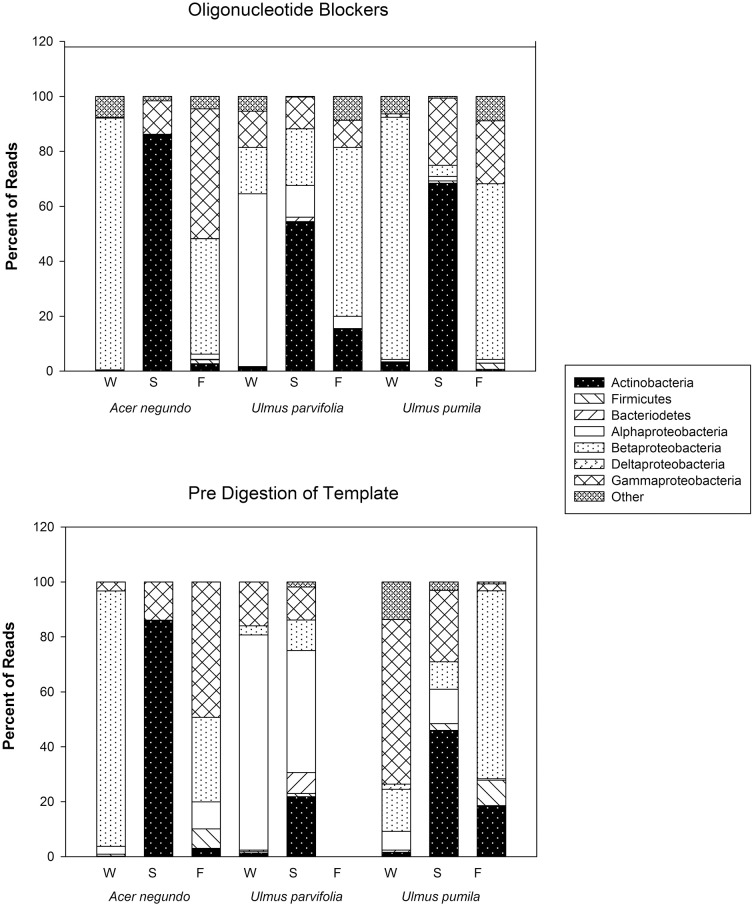
**Bacterial phyla, and class for Proteobacteria, and their relative abundances found in the amplicon sequences generated from pre-digested templates and from reactions involving the use of blocker oligonucleotides (see text)**.

The amount of reads that were obtained from the pyrosequencing of pooled samples varied between samples, as did the amount of reads that came from plant organelles. The latter averaged 41% when using predigested template DNA and 46% when using the blocker oligonucleotide method (Table 3). We attribute some sample to sample differences in the release of plastids from plant cells to seasonal differences in plant cell wall resistances in the extraction method. When sequences were grouped by phyla (class in case of proteobacteria), some differences between the two organelle avoiding techniques are seen (See Figure 4). Phylum/Proteobacterial Class assignments are highly correlated between the methods in seven of the 9 pooled samples analyzed, but there is poor correlation in two of the pools. We did not obtain enough reads from the Fall *U. parvifolia* digested template to perform analyses (Table 3). Where the correlations were poor, it was largely due to differing relative abundances of the proteobacterial classes. Both methods clearly show however that the Summer samples see high levels of Actinobacteria, while the Fall and Winter samples are dominated by Proteobacteria. Data have been deposited in Genbank under SRA Study number SRP055785.

**Table 3:** **Comparison of the 454 tagged pyrosequencing of the pooled samples from the same species and the same season, generated through either pre-digestion of the DNA using restriction enzymes (*Pvu*II and *Msc*I) or oligonucleotide blockers designed to inhibit the amplification of plastid and mitochondrial 16S rRNA**.

**Species**	**Season**	**Digest**	**Digest**	**Blocker**	**Blocker**	**Phyla**
		Reads	%Org	Reads	%Org	Correlation
*Acer negundo*	W	32260	86.75	24090	67.25	0.99
	S	20959	2.05	19744	2.3	1.00
	F	11789	82.35	16821	68.3	0.95
*Ulmus parvifolia*	W	4520	17.9	14121	13.35	0.97
	S	4752	22.05	12535	22.15	0.44
	F	11	−	21735	79.25	NA
*Ulmus pumila*	W	4520	32.7	19486	64.4	0.07
	S	3987	36.35	14330	14.8	0.96
	F	2299	49.45	22677	83.65	0.88

*The OTUs from both sample sets were rarified to 2000 when estimating organelle read content*.

**Figure 5:**
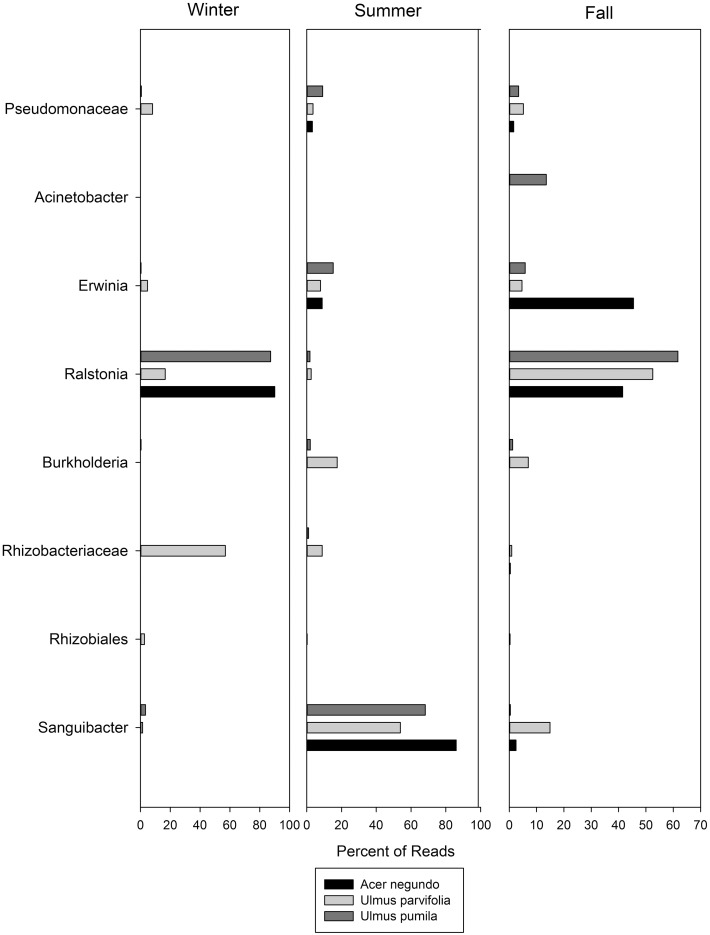
**The most abundant OTUs found in the pyrosequencing data along with their relative abundances through the seasons**. Names are given for the closest level of classification available in database.

The dominant OTUs found for each plant species in each season are shown in Figure 5, along with their relative abundances. In this figure OTU's are named at the highest level of classification obtainable, at the genus level where possible, but at family or order where not. Of particular interest is the dominance of *Sanguibacter sp*. in all three species during the summer. When an *in silico* digest to mimic T-RFLP was carried out using a python script on the OTUs obtained from the pyrosequencing, there were some OTUs that matched the dominant T-RFLP phylotype fragments. These included OTUs that corresponded to bacteria from the order Rhizobiales (T-RFLP fragment 128 bp), and *Ralstonia* spp. (T-RFLP fragment 488 bp).

## Discussion

It is clear from this work that the main determinant of the endophyte community structures in these tree species is the season. Our work involved the study of both culture dependent and independent methods and shows the latter to be more sensitive to seasonal differences (Table 2). Studies on the leaf phyllosphere of *Asclepias viridis* (Ding et al., 2013), the endophytes of maple tree sap (Filteau et al., 2010) and the buds of Scots pine trees (Pirttilä et al., 2005) have also noted a strong seasonal effect. The diversity of bacteria in the grape endosphere has been shown to be highly dependent on season (Baldan et al., 2014; Bulgari et al., 2014). In these studies, the research has involved the use of a variety of methods including T-RFLP for the study of the endophytes in leaves, PCR fingerprinting for the study of maple tree sap and 16S rRNA specific probes for the study of the buds of Scots. There is another study that has looked at variation of root and stems of 2 elm species (*U. japonica* and hybrid “Lobel”,) which corroborates with our research (Mocali et al., 2003) even though it was entirely based on culturable endophytes.

Seasonal community changes could be due to temperature optima of the bacteria or the changing physiology of these temperate deciduous trees which reflects their respective growth phases and external temperatures (Jansson and Douglas, 2007). Changes in the concentrations of soluble sugars, proteins, amino acids, organic acids, and other nutrients in the plant will affect the community (Cox and Stushnoff, 2001; Li et al., 2004; Renaut et al., 2005). Similarly, the carbon source in different plant species varies in the form of different nonreducing sugars, oligosaccharides, sugar alcohols, starch, and other polysaccharides that are present (Bloom et al., 1985). This would explain why after season, the tree genus impacts strongest on the endophytic community. It has also been suggested that the plant host plays an active role in the colonization of the endophytes by attracting specific bacteria, through the release of certain compounds via their roots (de Weert et al., 2002; Compant et al., 2005), or by enhancing or diminishing their colonization in the plant, through plant defense response and generation of phytohormones (Miché et al., 2006; Shah, 2009).

The effect of the tree genus on the endophytic community was clearly seen in the T-RFLP analysis of the endophytic community, but not in the culture collection. This reflects the drawbacks of the culture dependent method where bacteria do not all necessarily grow in the same type of media and a wide variety of them cannot be further isolated and purified. This might be why Izumi et al. (2008) did not see any differences between endophytic communities of three quite different tree species, as their methods relied on incubating plant material in TSB before plating on TSA. However, using four different media, Moore et al. (2006) were able to detect differences in the endophytes of hybrid Poplar cultivars growing intermingled on the same site. They also noted that no genotype (as determined by BOX fingerprints) existed in more than one tree zone (i.e., roots, rhizosphere, stems, and leaves) suggesting strong niche specialization in these bacteria. This supports previous studies that involved studying agricultural plant endophytes using culturable methods, where the plant genotype and cultivar affected the colonization of endophytes, such as in cotton plants (Adams and Kloepper, 2002), soybean (Okubo et al., 2009), and peas (Elvira-Recuenco and van Vuurde, 2000).

Even though different communities were detected through the use of molecular methods, further analysis of the T-RFLP profiles of the endophytes communities revealed the presence of 5 phylotypes common to all branches sampled regardless of season and plant species. These T-RFs could potentially represent a core group of bacteria phylotypes at this site. Mengoni et al. (2009) noted a very small group of T-RFLP fragments were found common amongst highly variable leaf-associated communities within one species. Carrell and Frank (2014) found that pine and spruce species in the same nutrient limited environment had species specific endophytes communities, but nonetheless shared a dominant diazotrophic *Gluconacetobacter* species. This does support the notion of both specific endophyte-plant relationships coexisting with more generalist endophytes that might confer special adaptive traits to their plant hosts.

The shared phylotypes seen in our study were detected as terminal restriction fragments generated based on polymorphisms specific only to *Msp*I cut sites in bacterial 16S rRNA. As one fragment (phylotype) in a T-RFLP analysis can result from more than one bacterial species or genera, we are likely underestimating diversity and also the number of possible shared bacterial genera (Abdo et al., 2006; Schütte et al., 2008). Based on the pyrosequencing data, these core bacterial phylotypes may correspond to *Pseudomonas* spp (although rare in the pyrosequence data), *Ralstonia* spp. and bacteria from the order Rhizobiales.

Culturing the endophytes on R2A and TSA resulted in the isolation of bacterial genera that have been previously been found in the internal tissues or phyllosphere of other crops and trees. In all the samples analyzed, the common presence of bacterial species from *Bacillus* spp., *Curtobacterium* spp., and *Sphingomonas* spp. was similar to for two elm species by Mocali et al. (2003). The majority of the identified cultured bacteria endophytes were classified under the phylum Actinobacteria, making up 62% and 63% of total bacterial isolated in R2A media and TSA media, respectively. The dominance of Actinobacteria has been previously seen in the study of endophytic communities including those of poplar trees (Ulrich et al., 2008), corn (Chelius and Triplett, 2001), wheat (Coombs and Franco, 2003), and *Arabidopsis thaliana* (Bulgarelli et al., 2012). Studies on Actinobacteria endophytes noted their roles as biological control agents (Coombs et al., 2004) and producers of novel natural products (Qin et al., 2012). In addition to these species we also isolated *Patulibacter* sp. which has been previously only been isolated from soil samples (Takahashi et al., 2006).

We were successful in revealing the dominant bacteria from these tree tissues by modifying our amplification procedures prior to pyrosequencing. Both the use of the predigested templates and organelle sequence blocking oligonucleotides were effective in yielding large numbers of bacterial reads. Pyrosequencing of amplicons from plant tissues without such template or primer modifications typically yields more than 90% organelle reads (Gottel et al., 2011; Lucero et al., 2011). However organelle reads were not eliminated by either method. For our predigestion method we speculate this was due to incomplete digestion caused by template abundance or methylation or perhaps to the amplification of organelle chimeras. In the case of the oligonucleotide blockers, their concentration might need optimization in the face of large amounts plant DNA. It is useful to note that the proportion of organelle sequences obtained by the two methods were significantly correlated (*r* = 0.76, *p* < 0.05), suggestive of a template effect (i.e., abundance of organelle targets). Nevertheless our work revealed the importance of some key genera to these trees.

The genus *Sanguibacter* was abundant in all three plant species in the Summer season. *Sanguibacter* is an Actinobacteria as were many of our isolates, but it did not appear in culture. Only two previous studies report *Sanguibacter* as an endophyte–Ulrich et al. (2008) cultured members of this genus from poplar trees and Mastretta et al. (2009) isolated some from seeds of *Nicotiana tabacum* growing at an industrial sewage sludge site. The *Sanguibacter* strain isolated from *N. tabacum* was found to be cadmium tolerant and when used to inoculate other *N. tabacum* plants exposed to different cadmium levels, it was found to increase the development of shoots and roots. Other than this, all *Sanguibacter* isolates have been reported from soils, sands, sediments or even blood and milk.

The pyrosequencing reads also revealed an abundance of Betaproteobacteria in the Winter and Fall samples with the majority of these OTUs corresponding to the genus *Ralstonia*. The *Ralstonia* genus is best known for including the wilt pathogen *R. solanacearum*, but our sequence data do not indicate that particular species was present. *Ralstonia* endophytes have been reported in grapevines (Campisano et al., 2014), in lettuce (Jackson et al., 2013) and in cowpea nodules (Sarr et al., 2010). The presence of the Gammaproteobacteria detected in the Summer and Fall corresponded to OTUs belonging to the genera *Erwinia* and unknown members of *Pseudomonaceae*. *Pseudomonas* spp. have been found to be make up the majority of genera analyzed in the maple sap microbiota (Mengoni et al., 2009) and also in the analysis of the poplar tree endophytes isolates (Moore et al., 2006). Some *Pseudomonas* are plant growth promoting strains (Kuklinsky-Sobral et al., 2004). However the role that each of these genera may play in the biology of these trees requires further research as ribosomal sequences can not reveal much about function, particularly where mobile elements might be involved.

Understanding the variation in the endophytic community allows for optimal sampling for the survey of endophytes and their capabilities in any vegetative community. The original choice of these trees for study was based on their location; they occupy an urban site contaminated by both petroleum and chlorinated solvents. However before investigating the potential role of these tree endophytes in any degradation of these organic contaminants, we needed to know how representative any isolated species might be. Now we know that the dominant strains can differ substantially over the seasons, and less so between species, we can better contextualize any beneficial functions these species might have. This study reveals that a better understanding of the potential role of *Sanguibacter* is warranted if we are to have insights into summer processes. Therefore it is necessary to study the endophytic communities of different types of plants through different seasons to fully appreciate the range of possible plant-endophyte interactions.

### Conflict of interest statement

The authors declare that the research was conducted in the absence of any commercial or financial relationships that could be construed as a potential conflict of interest.

## Supplementary material

Supplementary Figure 1 **Richness (number of species) and Shannon Diversity of culturable isolates obtained**.

Supplementary Figure 2 **Visual analysis of the culturable bacterial community profiles collected from R2A and TSA media**. Top plots: NMDS plot of the data obtained on R2A media, stress value = 0.343. Bottom plots: NMDS plot of data obtained on TSA media, stress = 0.350. Tree species symbols: °– *Acer negundo*, Δ– *Ulmus parvifolia* and + – *Ulmus pumila*. Season symbols: square – Winter, inverted triangle Summer and cross in box Fall.

Supplementary Figure 3 **Average number of phylotypes detected and calculated Shannon phylotypes diversity indices according to terminal restriction fragments obtained with forward labeled fragments and reverse labeled fragments, for each tree species and season**.

Supplementary Figure 4 **NMDS analysis of bacterial community profiles from the entire forward T-RFs dataset (stress value = 0.322); and from the entire reverse T-RFs data profiles (stress value = 0.346)**. Tree species symbols: Δ– *Acer negundo*, °– *Ulmus parvifolia* and + – *Ulmus* pumila. Season symbols: square – Winter, inverted triangle Summer and cross in box Fall.
