# Supplementary figures and images for: Seasonal variation of bacterial endophytes in urban trees
Source: Front Microbiol. 2015 May 19;6:427. doi: 10.3389/fmicb.2015.00427 (PMC4437045; doi:10.3389/fmicb.2015.00427)

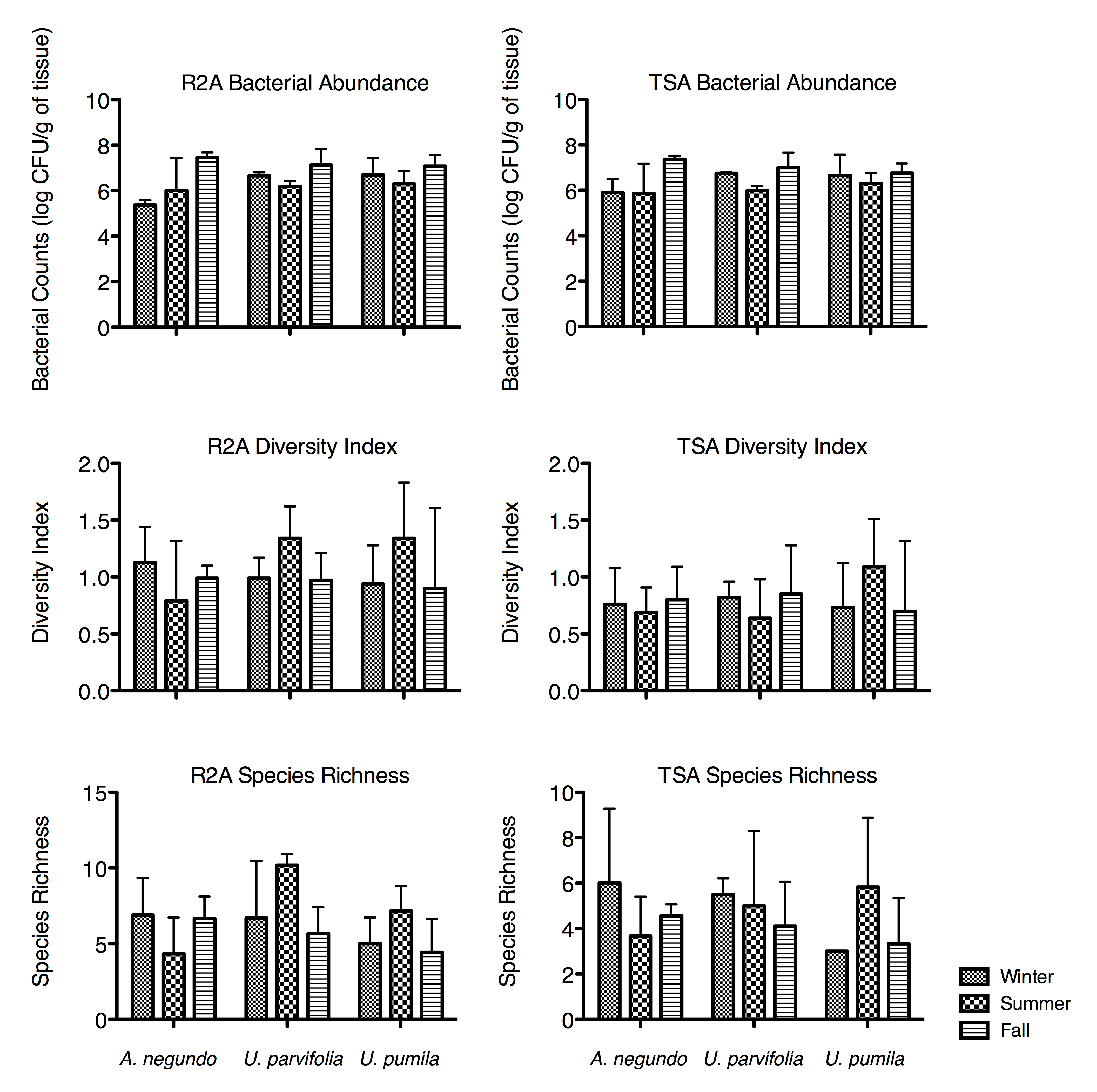

Supplement: Supplementary Figure 1 — Richness (number of species) and Shannon Diversity of culturable isolates obtained. [file Image1.JPEG]

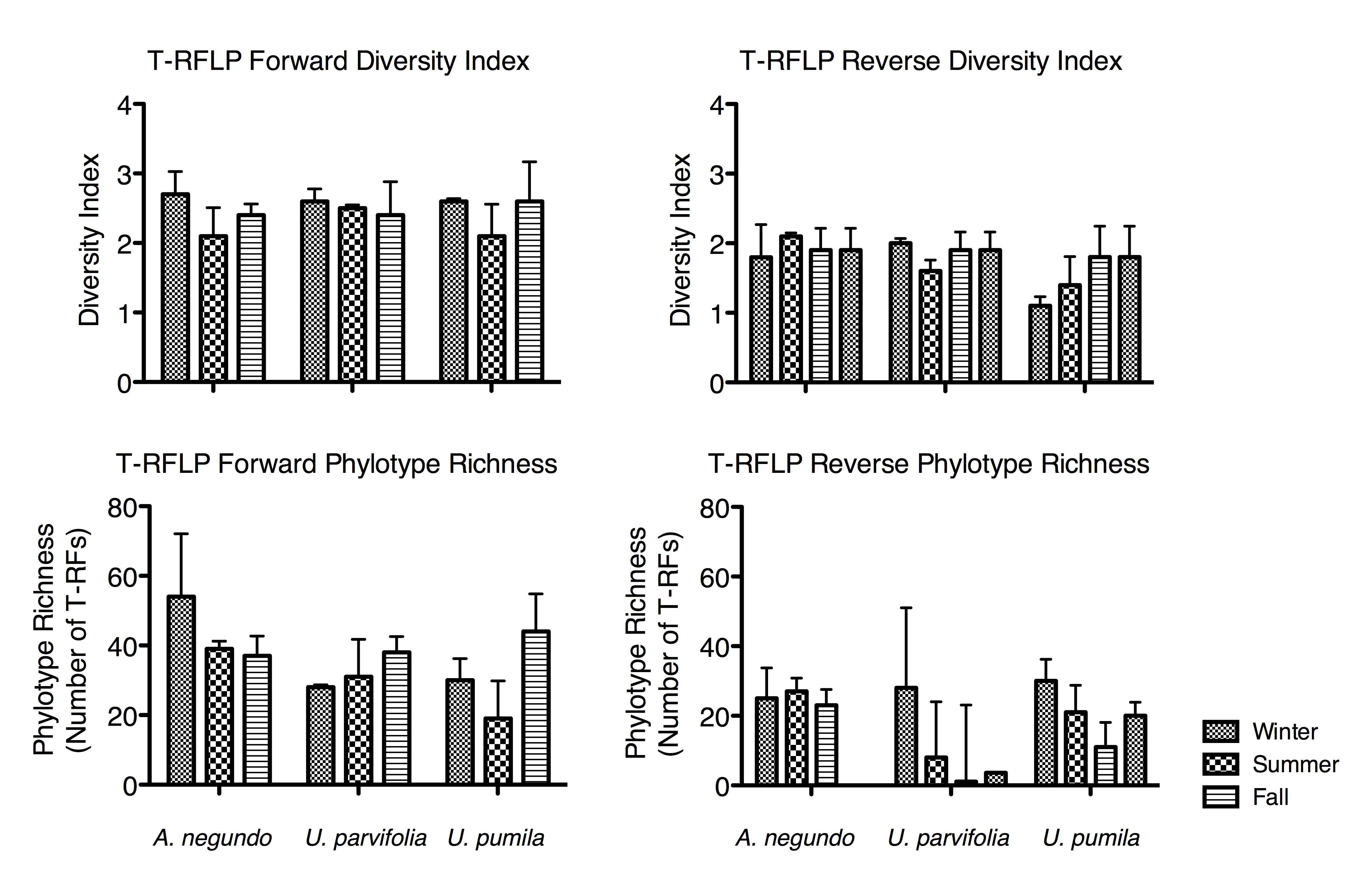

Supplement: Supplementary Figure 3 — Average number of phylotypes detected and calculated Shannon phylotypes diversity indices according to terminal restriction fragments obtained with forward labeled fragments and reverse labeled fragments, for each tree species and season. [file Image3.JPEG]
